# Albumin to gamma-glutamyltransferase ratio as a prognostic indicator in intrahepatic cholangiocarcinoma after curative resection

**DOI:** 10.18632/oncotarget.14530

**Published:** 2016-12-24

**Authors:** Chu-Yu Jing, Yi-Peng Fu, Hu-Jia Shen, Su-Su Zheng, Jia-Jia Lin, Yong Yi, Jin-Long Huang, Xin Xu, Juan Zhang, Jian Zhou, Jia Fan, Zheng-Gang Ren, Shuang-Jian Qiu, Bo-Heng Zhang

**Affiliations:** ^1^ The Liver Cancer Institute, Zhongshan Hospital and Shanghai Medical School, Fudan University, Key Laboratory for Carcinogenesis and Cancer Invasion, The Chinese Ministry of Education, Shanghai, P.R. China

**Keywords:** albumin, gamma-glutamyltransferase, intrahepatic cholangiocarcinoma, prognosis, nomogram

## Abstract

The prognosis of intrahepatic cholangiocarcinoma (ICC) remains poor whereas predictive models for survival prediction in ICC patients following curative resection are limited. Herein, we established a novel inflammation-based score derived from preoperative albumin to gamma-glutamyltransferase ratio (AGR) and evaluated its prognostic significance in ICC patients underwent curative resection. Prognostic value of AGR was retrospectively studied in a cohort comprising 206 ICC patients following curative resection. The predictive performance of AGR was compared with other inflammation-based scores and serological tumor markers in terms of concordance index (C-index). Further, prognostic nomograms incorporating AGR into the tumor-node-metastasis (TNM) staging systems were established to achieve a better discriminatory ability. The optimal cut-off value of AGR was 0.6. Multivariate analysis showed that AGR was an independent predictor for overall survival (OS; *P* = 0.003) and recurrence-free survival (RFS; *P* = 0.046). The C-index of AGR was superior to other inflammation-based scores and serological tumor markers in OS and RFS prediction. The established nomograms showed improved predictive accuracy compared with the TNM staging systems alone. These results indicate that AGR is an independent prognostic indicator for ICC underwent curative resection. The incorporation of AGR into the existing TNM staging systems achieved improved predictive accuracy.

## INTRODUCTION

Intrahepatic cholangiocarcinoma (ICC), marked by poor survival, is an epithelial malignancy arising from intrahepatic biliary tracts [1]. As a historically uncommon disease, ICC, which accounts for 5% to 10% of all cholangiocarcinoma [2], is attracting growing attention due to its steadily rising incidence and mortality across all continents [3]. Surgical resection is the only treatment potential for a cure [4]. However, even for patients underwent curative resection, survival remains dismal along with a 5-year survival rate of around 30% [5]. Therefore, clinically easy-accessible and individualized prognostic markers or scoring systems to stratify the prognosis in ICC patients following curative resection are urgently needed.

The commonly used staging systems for ICC were tumor-node-metastasis (TNM) staging systems, such as the American Joint Committee on Cancer (AJCC) seventh edition [6], the Liver Cancer Study Group of Japan (LCSGJ) system [7], the Okabayashi system [8] and the Nathan system [9]. Among the staging systems, issues on whether tumor diameter was an independent prognostic indicator, whether other non-TNM factors should be included and which staging system performed better in risk stratification remained controversial [9, 10]. Moreover, all these models were cumbersome and not specifically formulated for post-operative prognostic prediction.

Accumulating evidence showed that, on the basis of tissue damage, inflammation paved the way for carcinogenesis [11, 12]. The well-established risk factors of ICC, such as liver flukes, hepatolithiasis, primary sclerosing cholangitis, which caused chronic inflammations in the liver, indicated that inflammation was strongly correlated with the carcinogenesis of ICC [13]. In addition, studies based on our institutional data identified inflammation-based scores such as neutrophil to lymphocyte ratio (NLR), platelet to lymphocyte ratio (PLR) as prognostic factors in ICC [14, 15]. It is therefore reasonable to dig further into inflammation-based scores for the prognostic prediction in patients with ICC.

Gamma-glutamyltransferase (GGT) is an enzyme which is ubiquitously expressed on the surface of the epithelial cells that line glands and ducts. Historically, serum GGT level was a common indicator for hepatobiliary disease reflecting bile duct damage, the progression of liver cirrhosis and chronic hepatitis [16, 17]. Recent data suggested that higher serum GGT within the normal range was an early marker of oxidative stress and an indicator of higher cancer risk [18, 19]. Additionally, elevated serum GGT level was identified as an independent risk factor for poor prognosis in several cancer types, such as endometrial carcinoma and cervical cancer [20, 21]. Consistent with those studies, our previous studies proved elevated serum GGT level to be an independent predictor of poor survival in ICC and hepatocellular carcinoma (HCC) [22–24].

Albumin is a stable molecule synthesized by the hepatocytes which maintains the intravascular colloid oncotic pressure and transports various substances [25]. In clinic, hypoalbuminemia is a common indicator for malnutrition and liver dysfunction [26]. Under systemic inflammation, albumin serves as a protective agent that scavenges the reactive oxygen and nitrogen species, whereas the synthesis of albumin declined [26–28]. Previous studies showed that albumin alone or albumin-based markers were independent predictors of poor survival in several cancers [29–32]. In addition, a preclinical study demonstrated that albumin suppressed the proliferation of HCC cell lines [33]. Taken together, higher serum albumin levels were considered a protective factor for cancer patients.

As alluded to above, elevated GGT not only reflects hepatobiliary inflammation and underlying liver damage, but also is a marker of oxidative stress which indicates higher cancer risk and poor prognosis, whereas declined albumin level implies impaired liver function, malnutrition, severe inflammation and incompetency in eliminating oxidative stress. Hence, it is logic to propose albumin to gamma-glutamyltransferase ratio (AGR), a combination of liver function parameters which reflects the status of oxidative stress as well, as a novel inflammatory marker in the prognostic prediction for post-operative patients with ICC.

The goal of this study was to assess the prognostic value of AGR in patients with ICC following curative resection. Further, we aimed to compare the discriminative ability of AGR with other inflammation scores and conventional serological tumor markers to ascertain the feasibility of AGR as a prognostic indicator. Additionally, we tried to refine the existing staging systems by establishing a nomogram incorporating AGR into the existing TNM staging systems.

## RESULTS

### Clinicopathological profiles of the patients

The clinicopathological characteristics of the patients are detailed in Table 1. According to the AJCC manual 7th edition, the numbers of patients classified into stage I, II, III and IVa were 93, 60, 18 and 35, respectively. The numbers of patients classified into stage I, II, III and IVa based on LCSGJ staging system were 5, 93, 57 and 51, respectively. The median follow-up time was 18 months (range, 1–69 months). The 1-, 3-, 5-year OS and RFS rates were 73.1%, 49.1%, 38.0% and 55.2%, 32.1% 23.0%, respectively.

**Table 1:** Clinicopathological characteristics of patients with ICC: univariate and multivariate analysis

Variables	Patients (*n* = 206)	OS			RFS		
		Univariate *P*-value	Multivariate P-value	Multivariate HR (95%CI)	Univariate *P*-value	Multivariate *P*-value	Multivariate HR (95%CI)
Gender, male/female	126/80	0.960	NA		0.182	NA	
Age, years (median, range)	60, 28–85	0.800	NA		0.403	NA	
Liver cirrhosis, absent/present	170/36	0.320	NA		0.997	NA	
ALBI score, 1/2	151/55	0.106	NA		0.752	NA	
Child Pugh grade, A/B/unknown	192/6/8	0.737	NA		0.183	NA	
HBsAg, negative/positive	131/75	**0.027**	NS		0.135	NA	
Tumor size, ≤ 5/> 5cm	89/117	**0.022**	NS		**0.026**	NS	
Edmondson–Steiner classification, I–II/III–IV/unknown	170/30/6	0.761	NA		0.389	NA	
Tumor number, single/multiple	153/53	**< 0.001**	**< 0.001**	2.520 (1.641–3.872)	**0.001**	**0.006**	1.790 (1.183–2.708)
Direct invasion and local extrahepatic metastasis, no/yes*	184/22	**0.013**	NA		0.453	NA	
Lymph node metastasis, no/yes	171/35	**< 0.001**	**< 0.001**	2.978 (1.853–4.788)	**< 0.001**	**0.004**	1.974 (1.235–3.153)
MVI, no/yes	156/50	**0.004**	NS		**0.01**	**0.04**	1.539 (1.020–2.323)
**Conventional blood parameters**							
TBIL, ≤ 20.4/> 20.4 µmol/L	187/19	0.165	NA		0.906	NA	
Albumin, g/L (median, range)	41, 28 - 51	**0.008**	NS		0.833	NA	
Albumin, < 35/≥ 35 g/L	11/195	**0.007**	NS		0.332	NA	
GGT, ≤ 60/> 60 U/L	94/112	**< 0.001**	NS		**0.002**	NS	
ALT, ≤ 50/> 50 U/L	173/33	0.122	NA		**0.047**	NS	
ALP, ≤ 125/> 125 U/L	152/54	**0.022**	NS		0.099	NA	
CA19-9, < 37/≥ 37 U/L/unknown	97/104/5	**0.007**	NS		0.26	NA	
AFP, < 20/≥ 20 ng/mL/unknown	183/18/5	**0.042**	NS		**0.048**	NS	
CEA, < 5/≥ 5 ng/mL/unknown	160/41/5	**0.001**	NS		0.055	NA	
**Inflammatory parameters**							
GPR, ≤ 0.5/> 0.5	132/74	**0.001**	NS		**0.012**	NS	
AAPR, ≥ 0.5/< 0.5	66/140	**< 0.001**	NS		**0.003**	NS	
GAR, ≤ 3.5/> 3.5	120/86	**< 0.001**	NS		0.054	NA	
PNI, ≥ 45/< 45	169/37	**0.016**	NS		0.726	NA	
PLR, ≤ 175/>175	177/29	**0.012**	NS		0.472	NA	
NLR, ≤ 2.8/> 2.8	130/76	**< 0.001**	**0.004**	1.889 (1.222–2.919)	0.123	NA	
AGR, ≥ 0.6/< 0.6	108/98	**< 0.001**	**0.003**	2.011 (1.268–3.189)	**0.001**	**0.046**	1.477 (1.007–2.164)
**Conventional staging systems**							
LCSGJ stage, I–II/III/IVa	98/57/51	**< 0.001**	**NA**		**< 0.001**	**NA**	
AJCC 7th edition, I/II/III/IVa	93/60/18/35	**< 0.001**	**NA**		**< 0.001**	**NA**	

Abbreviations: OS, overall survival; RFS, recurrence-free survival; HBsAg, hepatitis B surface antigen; ALBI, albumin-bilirubin; MVI, microvascular invasion; TBIL, total bilirubin; ALT, alanine transaminase; GGT, gamma-glutamyltransferase; ALP, alkaline phosphatase; AFP, Alpha-fetoprotein; CEA, carcinoembryonic antigen; GPR, gamma-glutamyltransferase to platelet ratio; AAPR, albumin to alkaline phosphatase ratio; GAR, gamma-glutamyltransferase to alanine aminotransferase ratio; PNI, the prognostic nutritional index; PLR, platelet to lymphocyte ratio; NLR, neutrophil-to-lymphocyte ratio; AGR, albumin to gamma-glutamyltransferase ratio. *Direct invasion and local extrahepatic metastasis, included invasion of gallbladder, adrenal gland and diaphragm; LCSGJ, the Liver Cancer Study Group of Japan; AJCC, American Joint Committee on Cancer; *P*-value < 0.05 marked in bold font shows statistical significant.

### Relationship between AGR and patient characteristics

The median value of AGR was 0.64 (range, 0.03–3.0). The optimal cut-off value of AGR was 0.6 for survival prediction. Likewise, the cut-off values of other inflammation-based scores were listed in Table 1.

All these patients were classified into 2 groups via AGR: a low-risk group (AGR ≥ 0.6, *n* = 108) and a high-risk group (AGR < 0.6, *n* = 98). The clinicopathological characteristics for each group are listed in Table 2. The high-risk AGR group was presented with higher Child-Pugh grade (*P* = 0.01), higher albumin-bilirubin (ALBI) grade (*P* = 0.001) [34, 35], elevated alanine transaminase (ALT; *P* < 0.001), alkaline phosphatase (ALP; *P* < 0.001), carbohydrate antigen 19-9 (CA19-9; *P* = 0.001) and carcinoembryonic antigen (CEA; *P* = 0.008) levels, larger tumor size (*P* = 0.001), multiple tumors (*P* = 0.03), the presence of lymph node metastasis (*P* = 0.001) and advanced TNM stage (*P* < 0.001).

**Table 2:** Correlation between AGR and clinicopathological variables of patients with ICC

Variables	AGR ≥ 0.6 (n = 108)	AGR < 0.6 (n = 98)	P-value
**Gender, female/male**	49/59	31/67	**0.043**
**Age, years(median, range)**	61, 28–85	60, 35–79	0.722
**Liver cirrhosis, absent/present**	89/19	81/17	0.963
**ALBI score, 1/2**	90/18	61/37	**0.001**
**Child-Pugh grade, A/B/unknown**	105/0/3	87/6/5	**0.01**
**TBIL, ≤ 20.4/> 20.4 μmol/L**	102/6	85/13	0.056
**ALT, ≤ 50/> 50 U/L**	105/3	68/30	**< 0.001**
**ALP, ≤ 125/> 125 U/L**	100/8	52/46	**< 0.001**
**Albumin, < 35/≥ 35 g/L**	3/105	8/90	0.086
**GGT, ≤ 60/> 60U/L**	94/14	0/98	**< 0.001**
**CA19-9, < 37/≥ 37U/L/unknown**	63/43/2	34/61/3	**0.001**
**AFP, < 20/≥ 20 ng/mL/unknown**	97/9/2	86/9/3	0.807
**CEA, < 5/≥ 5ng/mL/unknown**	92/14/2	68/27/3	**0.008**
**Tumor size, ≤ 5/> 5cm**	59/49	30/68	**0.001**
**Edmondson–Steiner classification, I–II/III–IV/unknown**	92/14/2	78/16/4	0.601
**Tumor number, single/multiple**	87/21	66/32	**0.03**
*Direct invasion and local extrahepatic metastasis, absent/present	100/8	84/14	0.11
**Lymph node metastasis, absent/present**	99/9	72/26	**0.001**
**MVI, absent/present**	85/23	71/27	0.296
**AJCC 7th edition, I/II/III/IVa**	65/26/8/9	28/34/10/26	**< 0.001**
**LCSGJ stage, I–II/III/IVa**	67/29/12	31/28/39	**< 0.001**

Abbreviations: AGR, albumin to gamma-glutamyltranferase ratio; ALBI, albumin-bilirubin; TBIL, total bilirubin; ALT, alanine transaminase; ALP, alkaline phosphatase; AFP, alpha-fetoprotein; CEA, carcinoembryonic antigen; MVI, microvascular invasion; AJCC, American Joint Committee on Cancer; LCSGJ, the Liver Cancer Study Group of Japan; .*Direct invasion and local extrahepatic metastasis, included invasion of gallbladder, adrenal gland and diaphragm. *P*-value < 0.05 marked in bold font shows statistical significant.

### Prognostic significance of AGR

**Figure 1:**
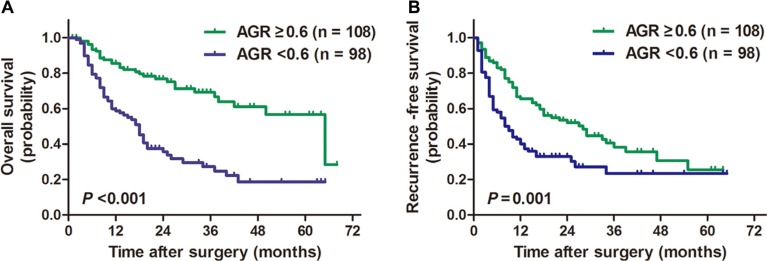
Kaplan-Meier survival curves for patients with ICC stratified by AGR ICC patients with a preoperative AGR lower than 0.6 were associated with significantly poorer overall survival (**A**) and recurrence-free survival (**B**) compared with ICC patients with a preoperative AGR larger than 0.6.

AGR less than 0.6 was associated with significant poor prognosis in terms of OS and RFS (*P* < 0.001 for OS; *P* = 0.001 for RFS; Figure 1). The 1-, 3- and 5-year OS rates for low-risk AGR group and high-risk AGR group were 85.4%, 69.0%, 56.1% and 59.7%, 26.9%, 18.1%, respectively. The 1-, 3- and 5-year RFS rates for low-risk AGR group and high-risk AGR group were 66.6%, 37.9%, 25.3% and 42.5%, 22.9%, 22.9%, respectively.

In univariate analysis for OS, Hepatitis B surface antigen positive (HBsAg; *P* = 0.027), larger tumor size (*P* = 0.022), multiple tumors (*P* < 0.001), presence of direct invasion and local extrahepatic metastasis (*P* = 0.013), lymph node metastasis (*P* < 0.001), microvascular invasion (MVI) (*P* = 0.004), declined serum albumin (*P* = 0.008) level, elevated serum GGT (*P* < 0.001), ALP (*P* = 0.022), CA19-9 (*P* = 0.007), alpha-fetoprotein (AFP; *P* = 0.042) and CEA (*P* = 0.001) levels, GGT to platelet ratio (GPR; *P* = 0.001) [17], albumin to ALP ratio (AAPR; *P* < 0.001) [29], GGT to ALT ratio (GAR; *P* < 0.001) [23], prognostic nutritional index (PNI; *P* = 0.016) [36], PLR (*P* = 0.012), NLR (*P* < 0.001), AGR (*P* < 0.001) and advanced TNM stage (*P* < 0.001 for both AJCC 7th edition and LCSGJ stage) were identified as significant predictors (Table 1). In multivariate analysis for OS, multiple tumors (*P* < 0.001, hazard ratio [HR] = 2.520; 95% confidential interval [CI] 1.641–3.872), presence of lymph node metastasis (*P* < 0.001, HR = 2.978; 95%CI 1.853–4.788), elevated NLR level (*P* = 0.004, HR = 1.889; 95%CI 1.222–2.919) and declined AGR (*P* = 0.003, HR = 2.011; 95%CI 1.268–3.189) level remained as independent indicators for OS.

In univariate analysis for RFS, larger tumor size (*P* = 0.026), multiple tumors (*P* = 0.001), presence of lymph node metastasis (*P* < 0.001) and MVI (*P* = 0.01), elevated ALT (*P* = 0.047), GGT (*P* = 0.002) and AFP (*P* = 0.048) levels, elevated GPR (*P* = 0.012), declined AAPR (*P* = 0.003) and AGR (*P* = 0.001) levels, and advanced TNM stage (*P* < 0.001 for both AJCC 7th edition and LCSGJ stage) were identified as risk factors for recurrence. In multivariate analysis for RFS, multiple tumors (*P* = 0.006, HR = 1.790; 95%CI 1.183–2.708), presence of lymph node metastasis (*P* = 0.004, HR = 1.974; 95%CI 1.235–3.153), MVI (*P* = 0.04, HR = 1.539; 95%CI 1.020–2.323) and decreased AGR (*P* = 0.046, HR = 1.477; 95%CI 1.007–2.164) remained as independent predictors.

To explore whether liver cirrhosis and hepatic function can affect the prognostic performance of AGR, subgroup analyses were performed. In ICC patients without liver cirrhosis, AGR can stratify both OS (Supplementary Figure 1A; *P* < 0.001) and RFS (Supplementary Figure 1C; *P* = 0.001). In ICC patients with liver cirrhosis, AGR was a prognostic indicator for OS (Supplementary Figure 1B; *P* = 0.002) but not for RFS (Supplementary Figure 1D; *P* = 0.239). Moreover, as illustrated in Supplementary Figure 2, AGR can stratify the OS and RFS in both ALBI grade I patients (*P* < 0.001 for OS; *P* = 0.002 for RFS) and ALBI grade II patients (*P* = 0.023 for OS; *P* = 0.04 for RFS). Taken together, AGR remained a prognostic indicator in ICC patients with different grades of hepatic function and liver cirrhosis.

### Comparative performance of AGR and other predictive models

**Figure 2:**
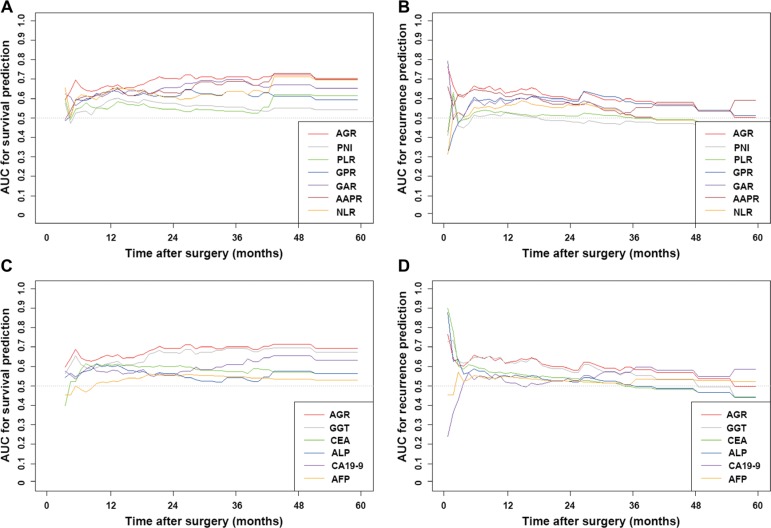
The time-dependent ROC curves of AGR, other inflammation-based scores and conventional blood parameters in death (**A** and **C**) and recurrence (**B** and **D**) prediction. Compared with other inflammation-based scores and serological tumor markers, the AUROC of AGR was the highest for death and recurrence prediction for the most of the follow-up time.

The discriminatory capabilities of AGR, other inflammation-based scores, serological tumor markers and conventional staging systems evaluated by concordance index (C-index) were shown in Table 3. The C-indices of AGR for OS and RFS prediction were 0.646 (95%CI 0.638–0.653) and 0.600 (95%CI 0.594–0.606), respectively. In terms of discriminatory capability, AGR outperformed other inflammation-based scores and conventional serological tumor markers in both OS and RFS prediction. Time-dependent receiver operating characteristic curve (ROC) showed that, compared with other inflammation-based scores and serological tumor markers, the area under the receiver operating characteristic curve (AUROC) of AGR was the highest for OS and RFS prediction for the most of the follow-up time (Figure 2; Supplementary Table 1).

**Table 3:** Discriminatory capabilities of staging systems and blood parameters in patients with ICC: C-indices in OS and RFS prediction

Variables	OS		RFS	
	C-index	95%CI	C-index	95%CI
**Combined predictive models**				
** Nomogram (AJCC 7th edition + AGR)**	0.736	0.730–0.743	0.650	0.643–0.657
** Nomogram (LCSGJ + AGR)**	0.731	0.724–0.738	0.658	0.651–0.664
**Staging systems**				
** AJCC 7th edition**	0.702	0.694–0.709	0.621	0.614–0.627
** LCSGJ stage**	0.699	0.692–0.705	0.633	0.626–0.640
**Inflammation based scores**				
** AGR (≥ 0.6/< 0.6)**	0.646	0.638–0.653	0.600	0.594–0.606
** GAR (≤ 3.5/> 3.5)**	0.614	0.606–0.624	0.553	0.549–0.559
** AAPR (≥ 0.5/< 0.5)**	0.608	0.601–0.614	0.522	0.517–0.527
** NLR (≤ 2.8/> 2.8)**	0.599	0.592–0.606	0.538	0.531–0.545
** GPR (≤ 0.5/> 0.5)**	0.598	0.590–0.605	0.559	0.553–0.565
** PNI (≥ 45/< 45)**	0.555	0.549–0.562	0.501	0.493–0.509
** PLR (≤ 175/> 175)**	0.545	0.539–0.550	0.514	0.509–0.519
**Conventional blood parameters**				
** GGT (≤ 60/> 60U/L)**	0.621	0.613–0.628	0.599	0.593–0.605
** CEA (< 5/≥ 5ng/mL)**	0.579	0.571–0.585	0.555	0.548–0.561
** ALP (≤ 125/> 125U/L )**	0.567	0.560–0.574	0.549	0.543–0.555
** CA19-9 (< 37/≥ 37U/L)**	0.564	0.556–0.572	0.522	0.514–0.529
** AFP (< 20/≥ 20ng/mL)**	0.526	0.522–0.530	0.530	0.526–0.534

Abbreviations: OS, overall survival; RFS, recurrence-free survival; LCSGJ, the Liver Cancer Study Group of Japan; AJCC, American Joint Committee on Cancer; AGR, albumin to gamma-glutamyltransferase ratio; GAR, gamma-glutamyltransferase to alanine aminotransferase ratio; AAPR, albumin to alkaline phosphatase ratio; NLR, neutrophil to lymphocyte ratio; GPR, gamma-glutamyltransferase to platelet ratio; PNI, the prognostic nutritional index; PLR, platelet to lymphocyte ratio; GGT, gamma-glutamyltransferase; CEA, carcinoembryonic antigen; ALP, alkaline phosphatase; AFP, Alpha-fetoprotein. The staging systems, inflammation based scores and other blood parameters were ranked based on C-index of OS prediction.

The conventional TNM staging systems were superior to inflammation-based scores and serological tumor markers in both OS and RFS prediction. The C-indices for OS and RFS prediction of the AJCC 7th edition were 0.702 (95%CI 0.694–0.709) and 0.621 (95%CI 0.614–0.627), respectively. The C-indices for OS and RFS prediction of the LCSGJ stage were 0.699 (95%CI 0.692–0.705) and 0.633 (95%CI 0.626–0.640), respectively. The predictive abilities of these two conventional TNM staging systems were not significantly different in our cohort.

### Prognostic nomograms integrating AGR and the conventional staging systems

**Figure 3:**
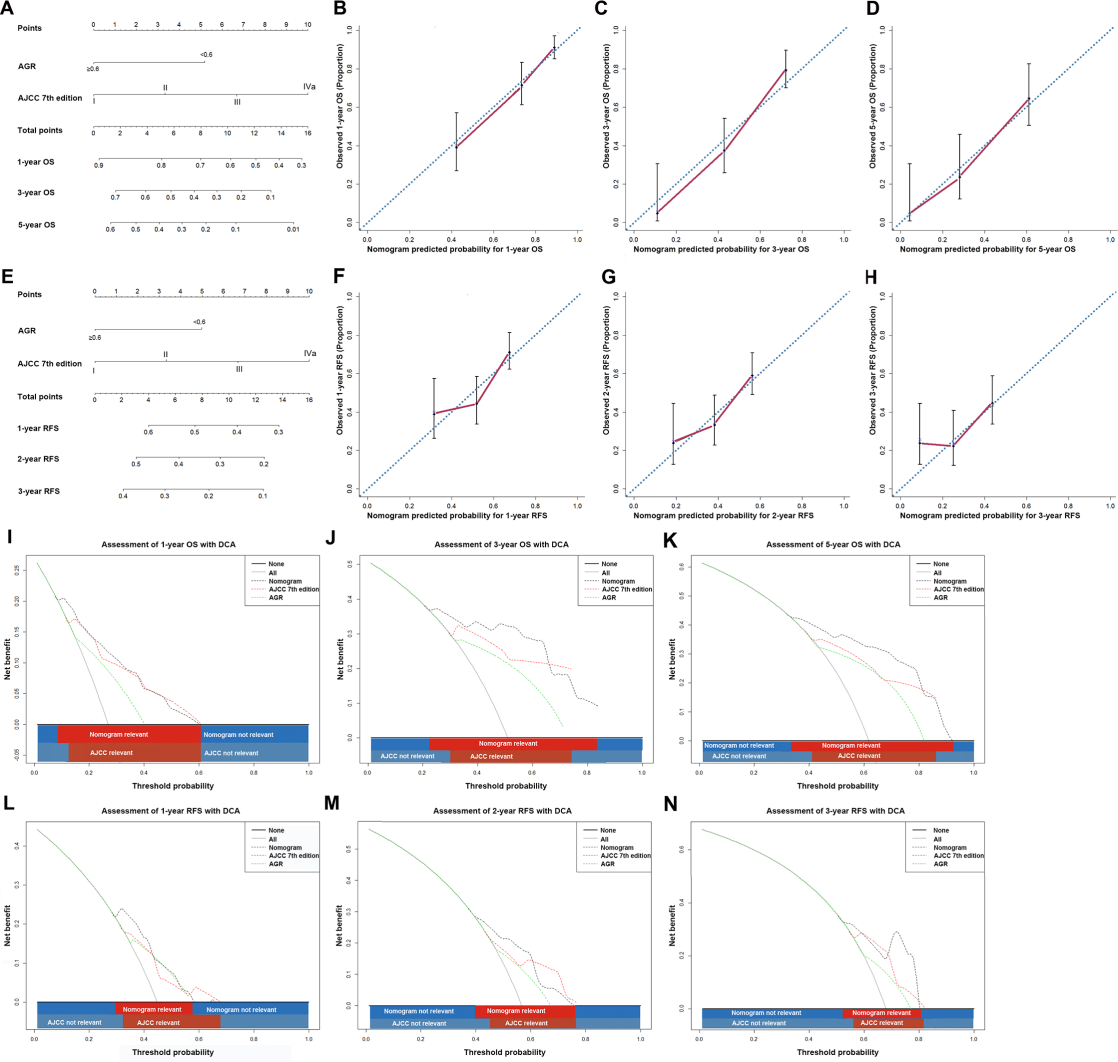
ICC prognostic nomograms, calibration curves and decision curve analysis Nomograms predicting (**A**) OS and (**E**) RFS in patients with ICC (to use the nomogram, an individual patient's value is located on each variable axis, and a line is drawn upwards to determine the number of points received for each variable value. The sum of these numbers is located on the Total Points axis, and a line is drawn downwards to the survival axes to determine the likelihood of 1-, 3- and 5-year OS. The calibration curves for predicting OS at (**B**) 1 years, (**C**) 3 years and (**D**) 5 years; predicting RFS at (**F**) 1 years, (**G**) 2 years and (**H**) 3 years. Nomogram-predicted probability of overall survival is plotted on the x axis and actual overall survival is plotted on the y axis. Decision curve analyses depict the clinical net benefit in pairwise comparisons across the different models. Nomogram is compared with the the AJCC 7th edition in terms of (**I**) 1-, (**J**) 3- and (**K**) 5-year OS and (**L**) 1-, (**M**) 2- and (**N**) 3-year RFS. Dashed lines indicate the net benefit of the predictive models across a range of threshold probabilities (black: nomogram; red: TNM stage; green: AGR). The horizontal solid black line represents the assumptions that no patient will experience the event, and the solid grey line represents the assumption that all patients will experience the event. On decision curve analysis, the nomograms showed superior net benefit compared with AJCC 7th edition across a wider range of threshold probabilities.

The nomograms integrating the AGR and the AJCC 7th edition for OS and RFS prediction gave rise to higher C-indices than that of the AJCC 7th edition alone (Table 3; Figure 3).

The C-index of the nomogram in OS prediction was 0.736 (95%CI 0.730–0.743) compared with the AJCC 7th edition with a C-index of 0.702 (95%CI 0.694–0.709). The calibration curves showed good consistency between the observed OS and nomogram-calculated OS at 1, 3, 5 years after surgery.

The C-index of the nomogram in RFS prediction was 0.650 (95%CI 0.643–0.657) compared with the previous value of 0.621 (95%CI 0.614–0.627) for AJCC 7th edition alone. The calibration plot for the probabilities of 1-, 2- and 3-year RFS fitted well between the actual observation and the prediction of the nomogram.

On decision curve analysis (DCA), a novel evaluation method that highlights the clinical net benefit of prediction models [37], the nomograms, compared with the AJCC 7th edition, yielded superior net benefit across a wider range of threshold probabilities. Likewise, the nomograms integrating the AGR and the LCSGJ stage for OS and RFS prediction yielded higher predictive power in terms of C-index (Table 3; Supplementary Figure 3).

## DISCUSSION

In this study, we established AGR, a novel, easily accessible inflammation-based score derived from preoperative serum albumin and GGT levels, as a predictor for survival of patients with ICC following curative resection. Next, we showed that AGR outperformed other inflammation-based scores and conventional blood parameters including serological tumor markers in terms of discriminatory capacity. Furthermore, nomograms incorporating AGR and TNM staging systems including the LCSGJ stage and AJCC 7th edition showed improved predictive power relative to the TNM staging systems alone.

Oxidative stress, a main product of inflammation, can cause DNA damage and plays an important role in carcinogenesis in liver cancers [38, 39]. Physically, GGT is crucial in maintaining the level of intracellular glutathione, which protects the cell from oxidative damage [27]. Elevated serum GGT was an indicator of oxidative stress [18]. Albumin, on the contrary, provides abundant anti-oxidative substances and is proposed as a protective factor for cancer patients [40]. Therefore, AGR was more than an combination of liver function tests, as historically considered, but also a reasonable proxy for anti-oxidant balance and, furthermore, a potential prognostic indicator. In accordance with previous studies and our hypothesis, after stratifying patients into 2 groups according to the optimal cut-off value of AGR, the subgroup with lower AGR (< 0.6) were associated with the following clinicopathological features: (1) higher Child-Pugh grade and higher ALBI score, which reflected impaired liver function; (2) raised ALT, ALP, which were also commonly as indicators of inflammation; (3) elevated tumor markers (CEA, CA19-9), larger tumor size, multiple tumors, the presence of lymph node metastasis and advanced clinical stage. Additionally, decreased AGR was identified as an independent risk factor for OS and RFS in this cohort.

In recent decade, a series of inflammation-based scores emerged as robust prognostic indicators in various malignancies [14, 32, 41]. To the best of our knowledge, the existing staging systems and predictive models for ICC, including the above mentioned TNM staging systems and two nomograms [42, 43], lacked indicators of systemic inflammation and liver function, which could offer additional information in prognostic evaluation. In addition, our previous study revealed that inflammation based scores strengthened the predictive power of conventional staging systems in HCC [44]. It is therefore logic to expect the incorporation of AGR to improve the predictive performance of the existing staging systems of ICC. Herein, we incorporated AGR into AJCC and LCSGJ staging systems and showed that AGR refined the predictive accuracy of AJCC and LCSGJ staging systems in terms of C-index. The results were supportive of the integration of AGR into conventional staging systems for an improved discriminative ability.

Several shortcomings of this study should be addressed. Firstly, the study was retrospective in nature and the patients enrolled herein were form a single institution of China, a country with different risk factors from western countries in the carcinogenesis of ICC [13]. Secondly, the study only contained patients underwent curative resection. Furthermore, due to the limited number of patients, an external validation was not performed. Therefore, future studies should be carried out to evaluate the prognostic significance of AGR in patients with different ethnic origin, advanced clinical stage and different treatment modalities.

In conclusion, these data suggested AGR, a novel and easy-accessible inflammation-based score, to be a robust indicator in prognostic prediction for ICC underwent curative resection. Furthermore, we confirmed that prognostic nomograms incorporating AGR into TNM staging systems provided improved predictive accuracy compared with the TNM staging systems alone.

## MATERIALS AND METHODS

### Patients

A total of 206 consecutive patients diagnosed with ICC presenting to Zhongshan Hospital, Fudan University and underwent curative resection from August 2005 to December 2014 were enrolled and retrospectively analyzed in this study. All of the enrolled patients met the inclusion criteria as follows: no preoperative anti-cancer treatments; no history and concurrence of other malignant tumors; no history of decompensation due to the liver cirrhosis; histologically proven ICC; complete removal of macroscopic tumors, local extrahepatic metastatic lesions and histopathologically confirmed negative resection margin larger than 1cm; complete clinicopathological and follow-up data; no infectious manifestation or history of inflammatory disease other than viral hepatitis. Cases with mixed cancers, tumor of uncertain origins or distant metastasis before the surgery were all excluded.

Laboratory tests including blood routine, albumin, ALT, GGT, ALP, AFP, CA19-9 and CEA were routinely performed within 3 days before the surgical resection. The hepatic function was assessed by the Child-Pugh classification and the ALBI grade [34]. The clinical staging was based on the AJCC 7th edition [6] and the LCSGJ staging system [7]. The histologic grade of the tumor was defined by the Edmondson–Steiner classification. This study was approved by the Ethics Committee of Zhongshan Hospital and informed consent was waived.

### Follow-up

Post-operative follow-up was carried out every 2 to 4 months after discharge as described in our previous study [24]. Serological tumor biomarkers, abdominal ultrasonography, and chest X-ray were routinely performed during each follow-up. Suspected recurrence or distal metastasis was validated by computed tomography and/or magnetic resonance imaging. The RFS time was defined as time interval between the date of surgery and the date when recurrence was first identified. The OS was calculated from the date of surgery to death. For patients without a documented RFS/OS event, the data were censored at the last follow-up.

### Statistical analysis

The AGR was calculated as the serum albumin level (g/L) divided by GGT level (U/L). Other inflammation-based scores including GAR [23], AAPR [29], NLR [15], PLR [14], PNI [36] and GPR [17] were calculated as previously described. The optimal cut-off values for AGR and abovementioned inflammation-based scores were determined using X-tile version 3.6.1 (Yale University, New Haven, CT, USA).

Statistical analysis was performed by SPSS version 21.0 (SPSS Inc., Chicago, IL, USA) and R project version 2.14.1 (http://www.r-project.org/). Differences between groups were analyzed using Pearson Chi-squared test, Fisher's exact test or Mann–Whitney U test as appropriate. The Cox proportional hazard model was used for both univariate analyses and multivariate analyses. The distributions of OS and the RFS were depicted by the Kaplan-Meier method and analyzed by the log-rank test. The discriminatory ability of AGR, other inflammation-based scores and serological tumor markers were evaluated by C-index and time-dependent AUROC. Time-dependent ROC was depicted using KM method via the survival ROC package in R.

Prognostic nomograms integrating traditional staging systems and the AGR were carried out by the rms package in R project. The performance of the nomogram was evaluated by C-index, calibration curve and the DCA as previously described [37].

## SUPPLEMENTARY MATERIALS FIGURES AND TABLE


