# Supplementary material for: Albumin to gamma-glutamyltransferase ratio as a prognostic indicator in intrahepatic cholangiocarcinoma after curative resection
Source: Oncotarget. 2016 Dec 24;8(8):13293–303. doi: 10.18632/oncotarget.14530 (PMC5355096; doi:10.18632/oncotarget.14530)
Supplement: Supplementary file 1 [file oncotarget-08-13293-s001.pdf]

Albumin to gamma-glutamyltransferase ratio as a prognostic indicator in intrahepatic cholangiocarcinoma after curative resection

Supplementary Materials

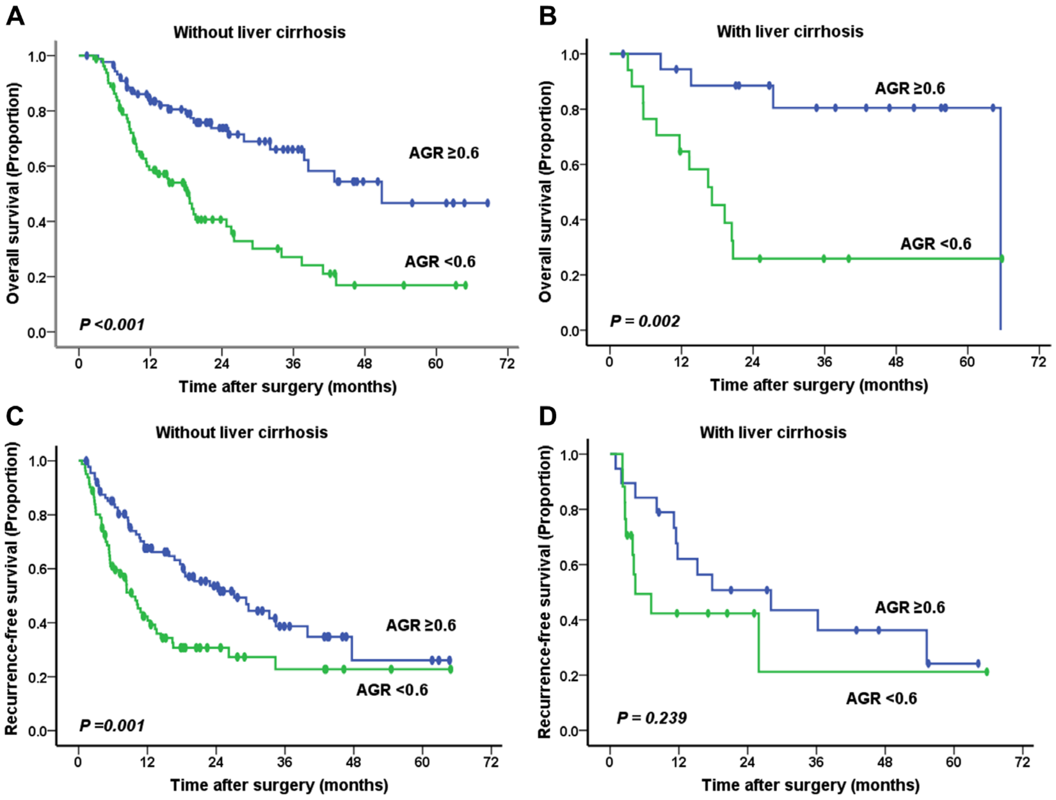

Supplementary Figure 1: Kaplan-Meier survival curves for patients with ICC patients with/without liver cirrhosis stratified by AGR. In patients without liver cirrhosis, AGR can stratify both OS (**A**) and RFS (**C**). In patients with liver cirrhosis, AGR was a prognostic indicator for OS (**B**) but not for RFS (**D**).

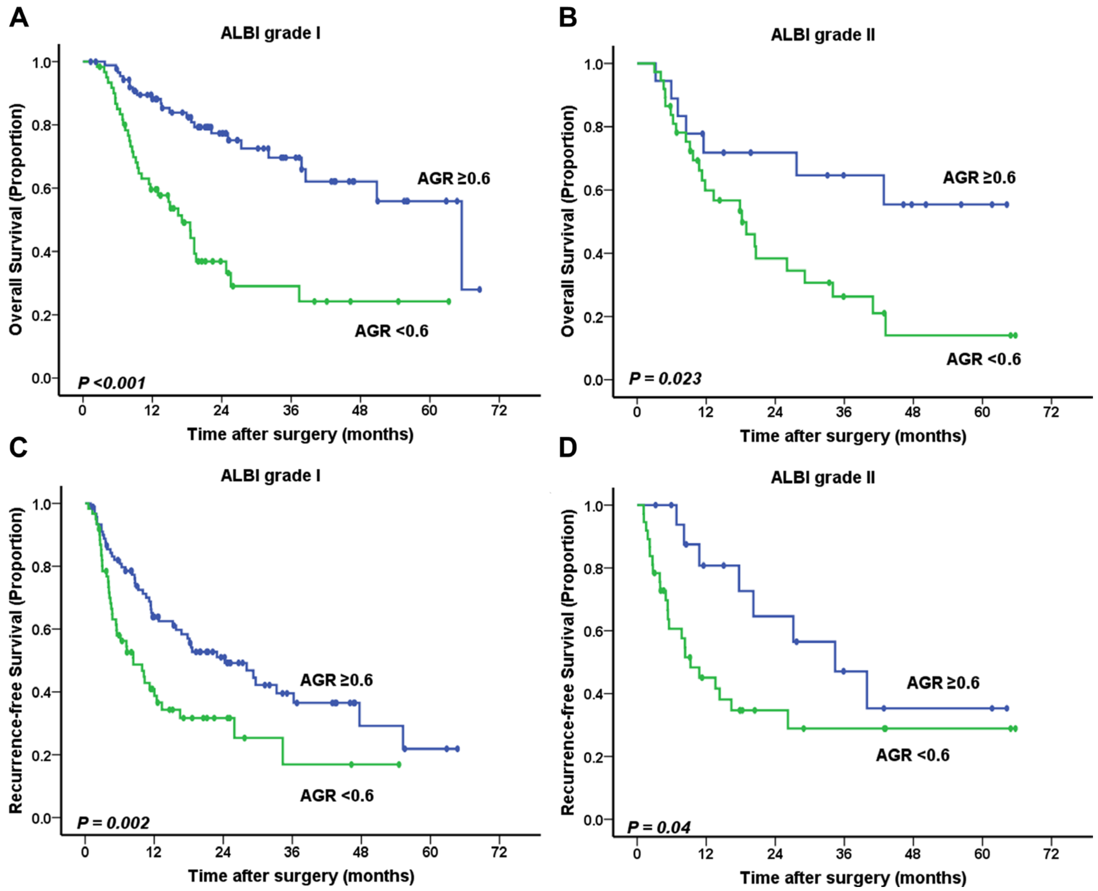

Supplementary Figure 2: Kaplan-Meier survival curves for patients with ICC patients with different ALBI grades stratified by AGR. In patients with different ALBI grades, the overall survival (**A** and **B**) and recurrence-free survival (**C** and **D**) between ICC patients with high and low AGR were significantly different.

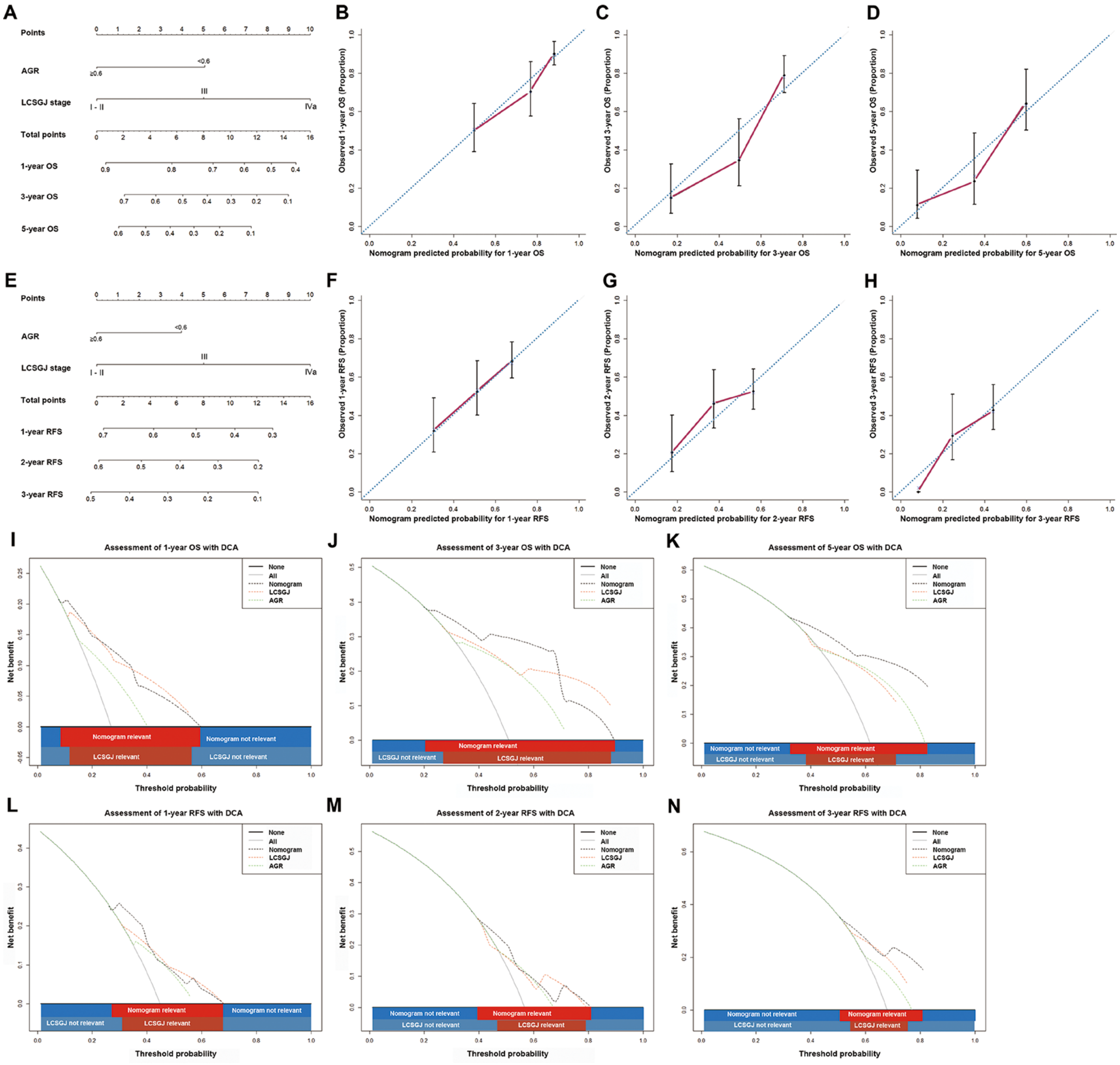

Supplementary Figure 3: ICC prognostic nomograms, calibration curves and decision curve analysis. Nomograms predicting (**A**) OS and (**E**) RFS in patients with ICC (to use the nomogram, an individual patient's value is located on each variable axis, and a line is drawn upwards to determine the number of points received for each variable value. The sum of these numbers is located on the Total Points axis, and a line is drawn downwards to the survival axes to determine the likelihood of 1-, 3- and 5-year OS. The calibration curves for predicting OS at (**B**) 1 years, (**C**) 3 years and (**D**) 5 years; predicting RFS at (**F**) 1 years, (**G**) 2 years and (**H**) 3 years. Nomogram-predicted probability of overall survival is plotted on the x axis and actual overall survival is plotted on the y axis. Decision curve analyses depict the clinical net benefit in pairwise comparisons across the different models. Nomogram is compared with the LCSGJ stage in terms of (**I**) 1-, (**J**) 3- and (**K**) 5-year OS and (**L**) 1-, (**M**) 2- and (**N**) 3-year RFS. Dashed lines indicate the net benefit of the predictive models across a range of threshold probabilities (black: nomogram; red: TNM stage; green: AGR). The horizontal solid black line represents the assumptions that no patient will experience the event, and the solid grey line represents the assumption that all patients will experience the event. On decision curve analysis, the nomograms showed superior net benefit compared with LCSGJ stage across a wider range of threshold probabilities.

Supplementary Table 1: AUROC for survival and recurrence prediction censored at different years after surgery in patients with ICC

Variables	AUROC for Survival					AUROC for Recurrence				
	1 year	2 years	3 years	4 years	5 years	1 year	2 years	3 years	4 years	5 years
AGR (≥ 0.6/< 0.6)	0.661	0.703	0.711	0.722	0.703	0.623	0.602	0.600	0.541	0.501
GAR (≤ 3.5/> 3.5)	0.639	0.656	0.700	0.670	0.653	0.566	0.579	0.518	0.459	0.416
AAPR (≥ 0.5/< 0.5)	0.650	0.630	0.687	0.728	0.699	0.615	0.581	0.527	0.536	0.592
NLR (≤ 2.8/> 2.8)	0.654	0.608	0.638	0.711	0.697	0.562	0.569	0.515	0.462	0.424
GPR (≤ 0.5/> 0.5)	0.637	0.609	0.637	0.611	0.595	0.588	0.592	0.584	0.541	0.511
PNI (≥ 45/< 45)	0.596	0.575	0.555	0.551	0.543	0.527	0.483	0.484	0.456	0.435
PLR (≤ 175/> 175)	0.560	0.545	0.536	0.619	0.614	0.529	0.511	0.501	0.481	0.466
GGT (≤ 60/> 60 U/L)	0.619	0.672	0.693	0.696	0.673	0.608	0.579	0.569	0.496	0.445
CEA (< 5/≥ 5 ng/mL)	0.607	0.599	0.573	0.572	0.563	0.560	0.540	0.497	0.466	0.443
ALP (≤ 125/> 125 U/L)	0.599	0.563	0.562	0.575	0.564	0.551	0.525	0.504	0.467	0.441
CA19-9 (< 7/≥ 37 U/L)	0.582	0.555	0.610	0.655	0.633	0.515	0.537	0.572	0.547	0.587
AFP (< 20/≥ 20 ng/mL)	0.526	0.554	0.549	0.534	0.531	0.548	0.526	0.507	0.528	0.522

Abbreviations: AUROC, area under the receiver operating characteristic curve; AGR, albumin to gamma-glutamyltransferase ratio; GAR, gamma-glutamyltransferase to alanine aminotransferase ratio; AAPR, albumin-to-alkaline phosphatase ratio; NLR, neutrophil-to-lymphocyte ratio; GPR, gamma-glutamyltransferase to platelet ratio; PNI, the prognostic nutritional index; PLR, platelet-to-lymphocyte ratio; GGT, gamma-glutamyltransferase; CEA, carcinoembryonic antigen; ALP , alkaline phosphatase; AFP, alpha-fetoprotein.
